# Quantitative Agent Based Model of User Behavior in an Internet Discussion Forum

**DOI:** 10.1371/journal.pone.0080524

**Published:** 2013-12-04

**Authors:** Pawel Sobkowicz

**Affiliations:** KEN 94/140, Warsaw, Poland; University of Warwick, United Kingdom

## Abstract

The paper presents an agent based simulation of opinion evolution, based on a nonlinear emotion/information/opinion (E/I/O) individual dynamics, to an actual Internet discussion forum. The goal is to reproduce the results of two-year long observations and analyses of the user communication behavior and of the expressed opinions and emotions, via simulations using an agent based model. The model allowed to derive various characteristics of the forum, including the distribution of user activity and popularity (outdegree and indegree), the distribution of length of dialogs between the participants, their political sympathies and the emotional content and purpose of the comments. The parameters used in the model have intuitive meanings, and can be translated into psychological observables.

## Introduction

Modeling of opinion dynamics using concepts from physics and computer simulations has a long history. There are numerous models designed to cover various aspects of individual and group influences and opinion changing psychological mechanisms. One should mention the voter model [1]–[4], the majority model of Galam [5], [6], the Sznajd model [7]–[14], the bounded confidence model [15]–[20], the Hegelsmann-Krause model [21], the social impact modef of Nowak-Latané [22], [23]. A general review is provided by Castellano et al. [24] or Galam [25]. These approaches have uncovered many interesting regularities in opinion dynamics of groups and social networks. Still, while these discoveries are interesting by themselves the simplifications and stylized nature of the descriptions of human behavior contained in the models makes their application to the actual social environments rather difficult.

On the other hand, there is a growing body of research that describes, in considerable detail, specific social environments (for example [26]–[29]). The natural next step in the development of *sociophysics* and opinion change simulations is, therefore, to build models that would be simple enough in their assumptions and parameters to remain understandable, yet would have the capability to correspond quantitatively to real-world situations. In the introduction to the recent special issue of the *Journal of Statistical Physics*, Fortunato, Macy and Redner [30] have observed that thanks to the increasing availability of rich datasets describing the human behaviors and interactions over the Web, the goal of **validating** the sociophysical models becomes feasible and desirable. An example of such application is a recent work combining analyses and simulation of user interests in online forums [31].

This work is an attempt in this direction for opinion dynamic studies: **it applies a general model to a specific social situation, with the goal of achieving not only qualitative but also quantitative agreement between the observed and simulated properties.** It is based on three foundations:

1. a detailed analysis of the properties of an Internet political discussion forum, including the social network statistics and extensive classification of the user political sympathies, types of comments and emotions expressed [32]–[34];
2. an agent based communication model, designed to reproduce the actual user behavior via a few intuitively understandable parameters (such as the probability of reading and writing a comment on a discussion forum, probability of entering into a quarrel with another user, etc.) [35];
3. a ‘microscopic’ model of individual opinion dynamics based on a nonlinear interplay of information available to the individual and his/her emotions [36].

The two latter models are combined together, with the goal of reconstructing as closely as possible the data gathered in the real forum. The reconstruction covers the statistical properties of the forum: the user in- and outdegree, the number of comments, the number and the size distribution of quarrels between users, the size of the giant connected component of the network formed by the comments. At the same time we want the model to reproduce reasonably well the ‘soft’ aspects of the forum: the distribution of political views (opinions) of the users, the emotional tone of the posts and the inferred emotions of their authors.

### Reference Social System: Political Internet Discussion Forum

Our work is based on extended, two year observations of two discussion fora connected with the most popular Polish newspaper, *Gazeta Wyborcza* [32]–[34]. The data gathered during the observed period included the social network structures given by the comments addressed to other forum users, statistical properties such as the distribution of indegree and outdegree, distributions of lengths of dialogs (usually quarrels) between pairs of users, lengths of discussions on specific subjects etc. Moreover, the analysis of the comment texts allowed to derive the political sympathies (treated as opinions) of the forum users and the comment emotional content. The comments were categorized according to their intent, namely as agreements/disagreements with other users, provocations, invectives and neutral postings.

It is important to note that the analyzed discussions, although related to the same news stories, took place in two separate Web environments, with marked difference in the easiness of forming dialogues between pairs of users. One version, historically first, therefore denoted as OLD, had a simple ‘reply to’ button and presented the comments in structure emphasizing the conversations, making it easy to participate in and to follow such pairwise exchanges. The other version of the forum, denoted hereafter as NEW, did not have this reply mechanism, but, on the other hand offered the possibility of evaluation of comments (thumbs-up/thumbs-down buttons), which did not require posting a comment. The absence of the automated reply mechanism in the NEW forum did not stop the users from creating dialogs, using in-line addressing. However, the tracing of the messages addressed to a user required a lot of attention. This difficulty led to a significant difference in the number of pairwise exchanges and in the size of the giant connected component of the social network. These differences were found to be correlated with statistically significant changes in the emotional character of the comments, with a greater ratio of strongly negative emotions in the NEW forum. As most of the dialogs were actually quarrels between the supporters of different political views, this finding was somewhat counter intuitive. The explanation offered by detailed analysis was that the writers of messages not addressed to a specific participant (broadcast messages) contributed strongly to these negative emotions. To grab the attention of the forum readers, the authors of these messages relied on expressing extreme opinions and abusive language.

## Model Description

### Message based Communication Network

The model presented in this paper aims to reproduce, using simple and realistic assumptions, the flow of messages between users. The proposed framework may, with some modifications be applied to diverse computer based communication environments: blog discussions, twitter messages, e-mails etc.

There are two basic entities in the model: agents and messages. The agents are characterized by a set of parameters, some of them static, some dynamically changing. These parameters characterize the basic user psychological characteristics (hidden from direct observation) as well as the measurable aspects of his/her behavior, such as the number of messages sent and received, publicly expressed opinion and emotional state, the social links with other agents etc. The agents perform three basic activities: reading of messages available (visible) to them, posting messages of various types and entering into extended dialogs (quarrels) with other agents, also via messages. All these activities are centered around the medium of the communication, we assume no direct, off-line contacts between the agents.

Depending on the modeled environment, there may be several kinds of messages: *directed* messages from one agent (*author*) to another (*target* or *recipient*) or to a group of *target* agents. These messages might be either *private* (i.e. accessible only to the author and the target agents, as is a typical situation for e-mail communications) or *visible* (while the message is addressed to limited group, it is visible to a broader range of agents, situation typical for discussion fora studied here). Lastly, the messages may be of the *broadcast* type, that is sent out by the author without specifying any target, with the goal of reaching wide audience. In some environments certain types of messages dominate, in others, there is a mixture of the types of the messages. There is also a possibility of *propaganda* messages, that is broadcast messages originated from outside the agent community, representing media (which may support one of the opinions and carry different amounts and valence of emotions).

As we have already mentioned, the studied environment consisted of two different interfaces, and as a result, some of the statistical properties of the discussions, most notably the emotional content of the messages turned out to be significantly different in the two environments. Despite these differences, there were some surprisingly common features for both versions of the discussion forum, which allowed us to propose a single parametrized model for both environments, and to look for the characteristic parameter values that correspond to the specific forum.

The agents (numbered by indexes *ai*, *aj* etc.) are characterized within the model by the following parameters.

- An intrinsic agent activeness (*A*(*ai*)), constant in time, which describes the general degree of agent willingness to participate in the forum activities (reading, writing messages etc.). *A*(*ai*) distribution in one of the inputs of the model.
- Actual, time dependent, agent activity and participation measures, such as its outdegree, the number of discussion threads the agent participated in, its indegree (measuring the interest its posts have generated or popularity).
- Agent internal state characteristics, for the opinion change model, including: *information* about the discussed topics (*I*(*ai*)); *opinion*, chosen to represent the political support for one of the two main conflicted political camps in Poland ((*O*(*ai*))); and *emotional state* (*E*(*ai*)). All these values are time dependent and may change due to reading of messages posted by other agents or propaganda messages. In the analyzed forum data to which the model would be compared, the opinions and emotions of participants were characterized by human coders, while the information available to the participant was, of course, not accessible to the observations.

### Network Construction for the Simulation

There are two options for constructing the communication network for the agent simulations of opinion and emotion changes. The first uses directly the cumulative data of the links between real users of the *Gazeta Wyborcza* forum. All messages generated in the simulation would follow the links in the imported network structure. This approach, while preserving the original structure (and additionally allowing to study the initial conditions in which simulated agents opinions and emotions are taken directly from the observations) has the disadvantage of being *too similar* to the original data. Thus, there is a danger that the simulations would be trivial and reproduce the particular behavior without providing an insight into the generality of the mechanisms involved in the communication processes and the opinion and emotion dynamics. Still, the approach could allow to study the system evolution on the arbitrary initial conditions, for example the formation of majority/minority balance.

The second approach is to build the communication network from scratch, using process elements aimed at reproducing realistically the flow of events in the real world. The simulated network would only be similar to the real one on a statistical basis. The goal of the network generation details is to reproduce as closely as possible, the key characteristics of the network: outdegree and indegree distribution, number and length distribution of the dialogs, number of broadcast and directed messages etc. Such approach would allow to study the consequences of individual behavior (the dynamics of opinion in the presence of influences and emotions) on the social system scale without relying on importing the specific network topology. Thus, the goal is to see if there are any general regularities in the whole process, combining personal changes and social communication. In the following work we have used this generative method of constructing the communication network.

The process of communication within the model may be described in the following steps:

1. An agent is chosen from the pool of all agents, with the probability 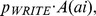 depending on the individual activity *A*(*ai*) and a global writing probability 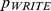. The agent sends a broadcast message, which reflects its opinion and emotional state, starting a discussion thread.
2. The message undergoes a reading loop, in which the program loops over all agents and performs the reading action with probability given by 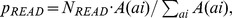 where 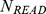 is a global parameter determining the average number a message is read). If an agent *aj* reads the message, its internal state of emotion information and opinion is updated, according to the individual dynamics, as described in the next Section. Then, with probability 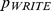 the reading agent has a chance to write a directed response, with the target being the author of the original broadcast message. Each new message generated in the reading loop is stored in program memory queue along with a time stamp. We note that there are more ‘reads’ than ‘writes’ in the model, exactly as is the situation in the real world.
3. Each message generated in step 2 is submitted to all agents for another reading. The behavior of the readers differs, depending on whether the agent is the target of the message or an outside onlooker:
  - The target agent process consists of a dialogue loop: state is updates and then the agent is ‘given a chance’ of responding in a user-to-user dialog with probability 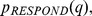 where *q* denotes the index of the message within the dialogue. If the response message is sent, it is submitted to the queue for reading by all agents, but immediately the roles of two agents (author/target) are exchanged and the new target agent is given the chance of replying. To preserve the statistics of dialogue lengths the probabilities of response depend on the number of message in the dialog *q*, the specific form of 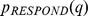 is described below. The quarrel loop ends when one of the pair of agents fails to respond.
  - The agents outside the quarreling pair treat all the messages generated within the quarrel as broadcast messages.
4. Once a quarrel loop is closed, the program returns to the reading process, starting with the oldest unprocessed message in the queue. The reading process is repeated until there are no new messages ‘generated’ by the response of readers, in which case the program goes back to step 1, starting a new thread.
5. The whole process ends when a predetermined number of messages is generated.

To construct the network that would share the basic properties with the actual Gazeta discussions we have made the following model assumptions:

- The distribution of the observed activity of the users in the forum (excluding the posts that form dedicated user-to-user dialogues), measured by the number of messages written follows with good accuracy, a power law form 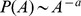. The exponents are very close to 2 for both the OLD and NEW interface, with the values of 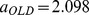 and 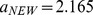. Therefore, the agent activeness *A*(*ai*), which is used to determine probabilities of all user activities (both writing the messages and reading, which is ‘invisible’ to the empirical analysis), is drawn randomly from the distribution following these power laws.
- The distribution of the observed dialogue (quarrel) lengths for both forums is well approximated by a lognormal distribution 
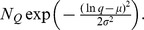
 The parameters of these distributions are significantly different for the different user interfaces: 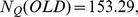 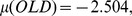 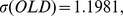 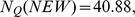 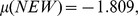 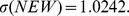 Such distributions means that the probability of *q^th^* reply within the dialog is not constant, but depends on *q*. The simulations use the successive probabilities of responding 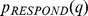 that reproduce the lognormal distributions characteristic for each forum interface.
- It should be noted that the values of 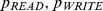 and all values of 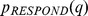, are rather small, which ensures that the characteristic ‘tree’ of messages resembles the actual discussion flow. In plain language: only a small number of agents from the whole population reads specific messages and even a smaller number writes new ones.

### User Behavior: Emotion, Information and Opinion (E/I/O) Model

The sociophysical models of opinion change mentioned in the Introduction usually treat individual opinions as the only changeable characteristic of the agents. In the simplest form, the opinion dynamics depends on the interactions between pairs of agents (or between an agent and a group of other agents), with the form of the interaction depending on the values of the opinions held by the agents. In fact, if one restricts the scope to the models treating the opinion as binary (+1/−1), many approaches may be treated as variants of a single framework [37]. In more advanced models the changes in opinions may also depend on some static characteristics of the agents, such as their power of influencing others or individual susceptibility to influence. These extensions are important in discussions of leadership [38]–[41]. Other static parameters are used to describe the role of inflexible agents [42], zealots [43], [44], inflexibles and contrarians [45]–[47] or independents and conformists [48]. The *ad hoc* introduction of such agents into the models has been necessitated by the need to reproduce such phenomena as persistence of conflict and minorities and other phenomena observed in real life.

In contrast to these works, the current paper is based on the E/I/O model proposed in [36], which extends the dynamical characteristics of an agent to include its emotion and information held about the issue in question. The goals of the model are very simple: to account for the observed individual psychological characteristics within a simple framework and to combine such individual description with a flexible communication mechanism allowing to use the model in different social contexts. Instead of introducing special classes of agents, we postulate that opinion dynamics of any agent, depends non-trivially on its information about the issue in question and on the emotional state, both of which may change itself as the result of inter-agent interactions or due to external sources such as media.

The model is a simplified version of the cusp catastrophe approach to opinion change [49], [50], with the information playing the role of the normal variable and emotion being the splitting variable. The simplification consists of the transition from continuous variables and the folded surface representing the allowed states, embedded in three-dimensional space of information, emotion and opinion, to a minimal discrete set of agent states located on this surface. There are seven states within the model, which may be described as using information and emotion as independent variables and opinion as the dependent one. With respect to emotions, we propose that agents may be either calm (C) or agitated (A). In the calm state, the agent’s opinion follows the available information about the issue in question which may take one of three states (+1, for example supporting party X, 0– neutral, and −1 – supporting party Y). The three calm states are thus described in the E/I/O model as CXX, C00 and CYY. We note here that in the calm state, changing the information available to an agent (e.g. via contact with another agent or through external sources, such as media or propaganda) can change the agent’s opinion. In other words, in the calm state, the agent is capable of rational evaluation of information and change of opinion.

The agitated states are characterized by the fact that the agent has a definitive opinion (either +1 or −1, with no ‘neutral’ option). There are two states in which the opinion is in agreement with the available information (AXX, AYY in the used notation). There are also two states for which, despite the lack of decisive information, an agitated agent keeps a well defined opinion (denoted as A0X, A0Y). These two states correspond to positions on the top and bottom part of the folded cusp catastrophe surface. The existence of these two states, in which the independent variables (emotion and information) take the same values, but the opinion may be different, depending on the history of the agent, makes the model non-trivial.

The next part of the model is the individual opinion dynamics. Each agent may change its state due to the influence of other agents and/or external media. In the following simulations we assume that such influence takes a form of separate messages (designed to mimic the discussion forum communication mode), each of which carries the emotional and informative content reflective of the state of the message author. The messages may either calm or agitate and change the information available to the reading agent. While there are many possible ways to describe the possible effects of the messages, the current paper is based on the transition table linking the original agent state, the message and the final agent state (Table 1).

**Table 1:** Matrix of states of agents resulting from a single message sent by the ‘Sender’ and received by the ‘Recipient’ in given state.

Recipient	Message content (state of the Sender)						
	CYY	C00	CXX	AYY	A0Y	A0X	AXX
**CYY**			AYY (*p_a_*)				
			C00 (1−*p_a_*)			AYY	A0Y
**C00**	CYY		CXX				
**CXX**	AXX (*p_a_*)						
	C00 (1−*p_a_*)			A0X	AXX		
**AYY**	CYY	CYY (*p_c_*)	A0Y (1−*p_c_*)				A0Y
			C00 (*p_c_*)				
**A0Y**	CYY	CYY (*p_c_*)	C00 (*p_c_*)				
**A0X**	C00 (*p_c_*)	CXX (*p_c_*)	CXX				
**AXX**	A0X (1−*p_c_*)	CXX (*p_c_*)	CXX	A0X			
	C00 (*p_c_*)						

Each cell is the final state of the recipient. For simplicity, only the changed recipient states are noted. In the case of CXX message received by a CYY agent (or vice versa) there are two possible outcomes: with probability 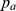, the recipient of the contrary message may get agitated, changing its emotional state from C(calm) to A(agitated), without changing the information nor the opinion, CYY→AYY. With probability 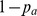, a calm contrarian message may convince the calm recipient to change its information and therefore, opinion, resulting in transition CYY→C00. Calm messages expressing the same opinion as the agitated agent holds, are assumed to always decrease its agitation due to the perceived ‘support’. On the other hand, a calm contrarian message may, with small ‘calming’ probability 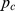, turn the agitated agent into calm neutral state C00.

The original model proposed in [36] was fully deterministic. In the current work we have introduced two exceptions: probabilistic parameters describing transitions between calm and agitated states. They correspond to intuitively understood reactions found in everyday life. The first of these parameters, the agitation probability 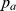, describes the irritability of an agent. Upon receiving calm message with contrarian views, instead of accepting the information and rationally re-evaluating its opinion, a calm agent may become irritated while keeping its opinion and change into the corresponding agitated state.

The second parameter, calming probability 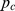, describes the opposite situation: with a small probability, an agitated agent may be calmed down by a neutral message. Similarly, with the same probability, a calm contrarian message may turn the agitated agent into a neutral (C00) one.

### Comment Types (Intent)

As we are interested in comparing the results of the model with the observations, we have also added to the model the rules determining the intent of the messages. By this we mean the intended meaning of the message with respect to its audience. For example the message may be a simple **neutral** statement of fact of opinion. It may be a calm **agreement** or **disagreement** with some previous message or agent. It may also be an angry message full of **invectives** rather than rational argumentation. Last category are **provocative** messages, written with the intent of angering their recipients. The intent of the message depends not only on the state of its author, but also on whether the message is addressed to a specific agent (and then on its state) or to the community at large.

It is very difficult for an agent based model to reach down to the level of single messages. In the real world, the intent of the author depends on many transient circumstances. The content may be interpreted in various ways, depending on the arguments used, wording, presence of sarcasm etc. Simulated agents are not sentient, we must remember that the model is ‘mechanistic’ in nature, and the type of the message ‘created’ by the agent is artificially assigned. In the simplest approach, we could assume that messages between a pair of agents would be of the same type (agreements, disagreements etc.) as long as the agents’ states do not change, for example messages between two CXX agent would always be agreements, those between CXX and CYY agents would always be disagreements, AXX and AYY agents would always exchange invectives, etc. The analysis of the forum datasets shows that such simplification is not true. Two corresponding CXX agents sometimes would write agreements and sometimes only neutral statements. Within a heated quarrel between two opposing agitated agents there are invectives and disagreements in varying proportions. The messages written to no-one in particular (broadcast messages) may be neutral or provocative.

To reflect these uncertainties, there are three probabilistic parameters that are used in simulations of the message intent. The first describes the probability of expressing agreement in a message between two agents sharing the same views. An agitated agent writing to another agent sharing the same opinion would write a message expressing agreement (AGR). However, a calm agent supporting one of two options, writing to another calm agent of the same opinion might either agree (with probability 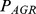) or write a neutral statement (with probability 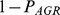). Similarly, a calm agent such as CXX or CYY, writing to a neutral C00 agent might either disagree with the neutral stance (probability 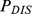) or write a neutral message (probability 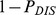). The agitated agents writing to the neutral C00 recipients have the options of writing provocative (PRV) messages or neutral ones neutral (with respective probabilities of 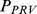 and 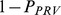). The same applies to broadcast messages by agitated agents. Lastly, an agitated agent writing to a calm opponent may either write a disagreeing message (DIS) or invectives (INV). The resulting process of assigning message type had therefore to be also probabilistic (Table 2).

**Table 2:** Table of message types resulting from a combination of Sender state and Recipient state, including messages not addressed to a specific user (broadcast messages).

	State of the Sender						
Recipient	CYY	C00	CXX	AYY	A0Y	A0X	AXX
**CYY**	AGR (*P_AGR_*)	NEU	DIS	AGR	AGR	DIS (*P_DIS_*)	DIS (*P_DIS_*)
	NEU (1−*P_AGR_*)					INV (1−*P_DIS_*)	INV (1−*P_DIS_*)
**C00**	DIS (*P_DIS_*)	NEU	DIS (*P_DIS_*)	PRV (*P_PRV_*)	PRV (*P_PRV_*)	PRV (*P_PRV_*)	PRV (*P_PRV_*)
	NEU (1−*P_DIS_*)		NEU (1−*P_DIS_*)	NEU (1−*P_PRV_*)	NEU (1−*P_PRV_*)	NEU (1−*P_PRV_*)	NEU (1−*P_PRV_*)
**CXX**	DIS	NEU	AGR (*P_AGR_*)	DIS (*P_DIS_*)	DIS (*P_DIS_*)		
			NEU (1−*P_AGR_*)	INV (1−*P_DIS_*)	INV (1−*P_DIS_*)	AGR	AGR
**AYY**	AGR	NEU	DIS	AGR	AGR	DIS (*P_DIS_*)	DIS (*P_DIS_*)
						INV (1−*P_DIS_*)	INV (1−*P_DIS_*)
**A0Y**	AGR	NEU	DIS	AGR	AGR	DIS (*P_DIS_*)	DIS (*P_DIS_*)
						INV (1−*P_DIS_*)	INV (1−*P_DIS_*)
**A0X**	DIS	NEU	AGR	DIS (*P_DIS_*)	DIS (*P_DIS_*)	AGR	AGR
				INV (1−*P_DIS_*)	INV (1−*P_DIS_*)		
**AXX**	DIS	NEU	AGR	DIS (*P_DIS_*)	DIS (*P_DIS_*)	AGR	AGR
				INV (1−*P_DIS_*)	INV (1−*P_DIS_*)		
**Broadcast**	NEU	NEU	NEU	PRV (*P_PRV_*)	PRV (*P_PRV_*)	PRV (*P_PRV_*)	PRV (*P_PRV_*)
				NEU (1−*P_PRV_*)	NEU (1−*P_PRV_*)	NEU (1−*P_PRV_*)	NEU (1−*P_PRV_*)

In most of the cases the message type (its intent) is fully determined by the Sender and Recipient states, for example CYY agent writing to CXX would write a disagreeing comment (DIS), AYY agent writing to another AYY would always agree (AGR). In some cases, however, the message type may be determined probabilistically. For example, a calm CYY agent writing to another calm CYY one may agree with probability 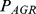 or simply write a neutral statement with probability 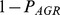. An agitated agent writing to an opponent (e.g. AXX writing to CYY or A0Y or AYY) may disagree with probability 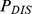 or abuse the opponent with invectives with probability 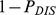. Lastly, while the broadcast messages of calm agents are always neutral (for broadcast messages there is obviously no possibility of agreeing or disagreeing), the broadcast messages of agitated agents may be provocations, with probability 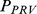.

## Results of Simulations

**Figure 1:**
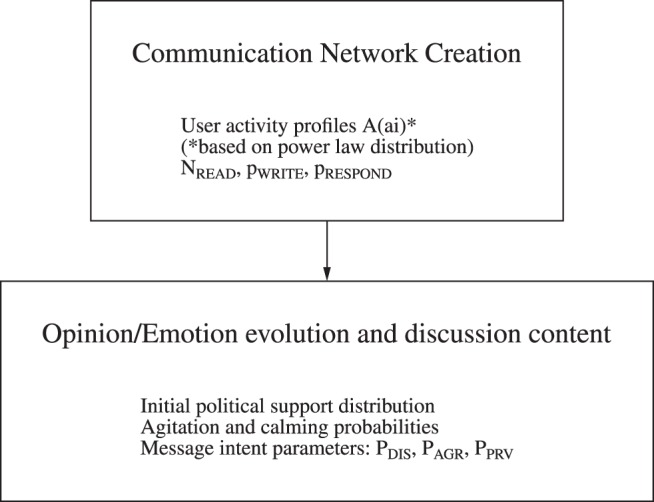
Block diagram of the two simulation subprograms. The first subprogram creates the communication network, using the listed parameters, the second provides the individual evolution of emotions and opinions as well as the intent of the messages exchanged in the discussions. For each set of parameters the network is generated between 10 and 25 times; then for each network instance the process of modelling the opinion ane emotion evolution is repeated 200 times.

For practical purposes, the computer program for the simulated environment was divided into two parts, as shown in Figure 1. The first part was used to create the comment network, taking as input parameters the user activity profiles *A*(*ai*), the profiles of dialog lengths given by 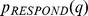, the average number of reads per comment 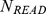 and the probability of writing a comment 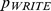. The total number of agents was assumed to be about 30–50% larger than the numbers of agents that actually participated in the discussions (the total number and 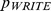 are connected via the number of generated messages). For each set of parameters, the program run several times to establish the average values of specific outcome parameters (such as the number of active users and broadcast messages), probability distributions of user in-degree, out-degree and the dialog lengths, and to determine the best values of the fitted parameters 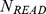 and 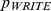. This part of the program produced the basic network structure of messages (author, target, time order) and of instances of agents reading the messages (whether resulting in writing a new comment or not). At this stage neither the agents nor the messages had any emotion, opinion or intent values.

The second part of the program used the ‘empty’ message, reading the timeflow and network structure to simulate the changes of opinion of individual agents and the message intent. The simulation started from some initial values of agents’ emotion, information and opinion and monitored the changes of opinions, emotions expressed in the comments as well as their intent type. Again, this process was repeated (200 times for each network configuration) to yield the average values of the final political party support, and comment type distributions. We have to stress here the significant role of the initial configurations for the simulation results.

The message network is ‘built from scratch’ with the same ‘empty’ starting conditions, so that the differences between subsequent runs (using the same parameters) come from randomness of the reading/writing process. On the other hand, the opinions (political affiliation) simulations pose a different type of the problem: how to distribute the initial values among the population of agents. In fact, this problem is quite common to all opinion dynamics models – and largely neglected.

Most of the agent based simulations of consensus formation start with a random distribution of initial agent opinions on the available ‘social space’ (which can be a network or a simple 2D array). The origin of this reliance on random starting state is easily traced to the physical origins of the sociophysical models. It should be noted however, that in reality, the distributions of people traits in social networks are seldom random. Assortative and disassortative social processes often produce nonrandom correlations, such as clusters of people with similar characteristics grouped together. Such groups show different temporal dynamics from the random configurations, even of the general ratios in the population are the same. Thus in many cases a pre-arranged, clustered starting configuration may show drastically different behavior than a random one. This problem has been addressed to some extent in [51].

Despite these reservations, we have decided to use random assignment of the initial agent states (emotions and opinions) in our model. This choice relates to the assumed initial lack of connections between the agents: the social network is *created* by the reading/writing process, but the forum users may come from diverse and uncorrelated environments. We assume that there are no coordinated activities by groups of like-minded agents, assumption based on the analysis of the original data.

The initial calm/agitated agent ratio, 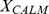, which can not be derived from the observations, is treated as fitting parameter, along with the agitation and calming probabilities defined in Table 1, 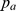 and 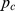. Similarly, the probabilities defining various types of messages, described in Table 2, 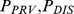 and 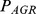 were fitted to obtain the best agreement with the observed forum characteristics. The final values of the best fit parameters are listed in Table 3.

**Table 3:** Best fit parameters used in reproducing the political affiliation, emotional content of messages and the type of messages.

	*p_a_*	*P_c_*	*X_CALM_*	*P_PRV_*	*P_DIS_*	*P_AGR_*
OLD	0.5	0.1	0.9	0.9	0.6	0.55
NEW	0.1	0.02	0.3	0.97	0.5	0.6

### Simulated Communication Network

**Figure 2:**
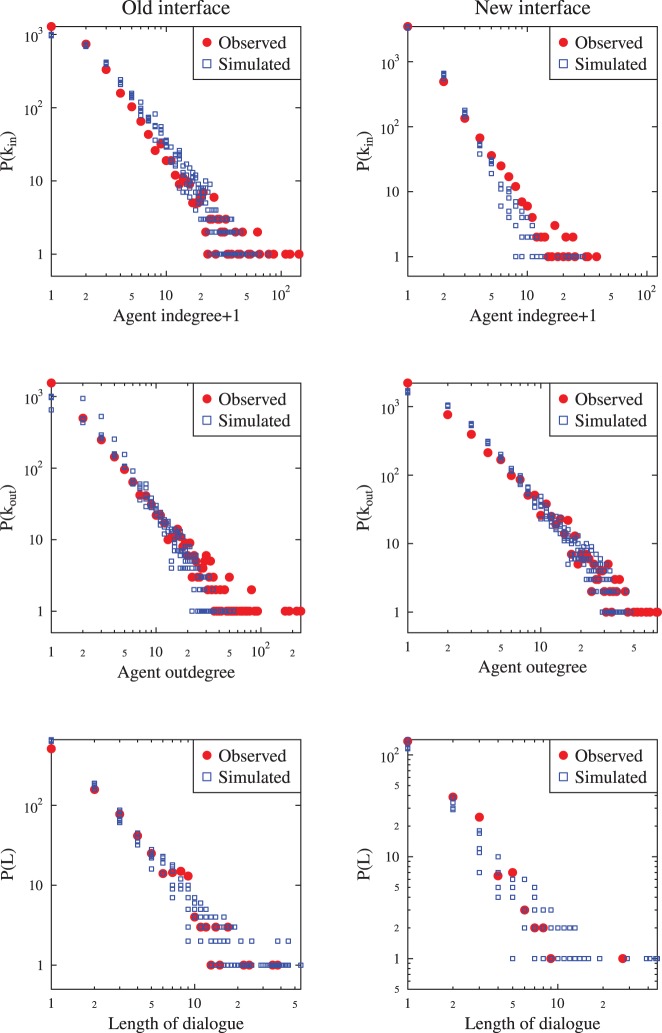
Comparison of basic network characteristics between the observations and simulations for the two discussion fora. The distribution of the agent in-degree, out-degree and the length of dialogs between pairs of users are presented. Blue squares represent results of five independent runs of the network simulation module, red dots are the observed values.

The optimal fit of the simulated network to the multiple characteristics of the observed network has been obtained using the following values of the starting parameters: the average number of reads per agent 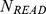 is 4 for the OLD forum, and 6 for the NEW one; the probability of writing 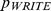 is 0.011 for the OLD forum and 0.012 for the NEW one. These two parameters, together with the overall number of users and the total number of messages (listed in Table 4) resulted in generally very good reproduction of the network characteristics such as the numbers of active users, numbers of broadcast messages and messages in inter-user dialogs (see Table 4). In addition we were able to reproduce the distributions of the user activity (outdegree) and popularity (indegree) as well as the distribution of the dialog lengths (Figure 2). The relatively small changes in the model parameters enabled to simulate strongly different environments of the OLD and NEW forum, related to the easiness of creating the dialogs and quarrels.

**Table 4:** Comparison of basic network parameters between the observed *Gazeta Wyborcza* fora and computer simulations.

	Old interface		New interface	
Network properties	Observed	Simulated	Observed	Simulated
Number of posts	12183	12104*	13631	13701*
Total number of users		4000*		6000*
Active users	2946	2941±33	4299	4276±45
Broadcast messages	4954	4373±97	11646	12242±67
Messages in dialogs	2314	2388±96	431	513±38

Asterisk * denotes values that are input of the simulations, other values are average results of multiple runs of the model, together with the standard deviation.

### Simulated Political Affiliation and Message Types

While simulations of the network structure were rather straightforward and led to good agreement with the observations, building the model of the content of the messages and the political breakup of the community was much more difficult.

The first problem was related to the initial ratio of political sympathies. The comment network, as described above, is built ‘from scratch’, in a selfconsistent way designed to mimic the actual reading/writing process. On the other hand, the Emotion/Information/Opinion dynamics model requires as the input the initial distribution of the agents’ political affiliations and emotions – before the agents start to leave trace in the discussions. We have chosen to use, as the initial values of the political sympathies of the agents, the data from the parliamentary elections that took place shortly after the studied period.

The second problem was related to the number of the political parties taken into consideration. Initially, we have attempted to follow the analyses presented in [32]–[34], which have focused on the conflict between two main political parties in Poland: PO and PiS. Unfortunately, it turned out to be impossible to achieve any reasonable fit to the observed user and message characteristics, especially with respect to the number of neutral (non-PO, non-PiS) agents. The dynamics resulting from the E/I/O model decrease the number of true neutrals very fast – contrary to the observations, where a significant number of agents were assigned to the neutral group.

To resolve this problem we have re-examined the analyses presented in [32]–[34], taking into account that Poland has a multi-party system. These works have already noted that the ‘neutral’ participant category comprises of two types of the users: the true neutrals without an opinion and supporters of any party other then PO or PiS. The parliamentary elections have shown a non-negligible minority of voters supporting the left-wing parties, SLD and RP (PO–40% of votes, PiS–29%, SLD/RP–19%). In fact, the comments in the *Gazeta Wyborcza* forum supporting the left wing parties were previously characterized as neutral from the point of view of the bipartisan conflict, because these users did not support PO nor PiS.

**Figure 3:**
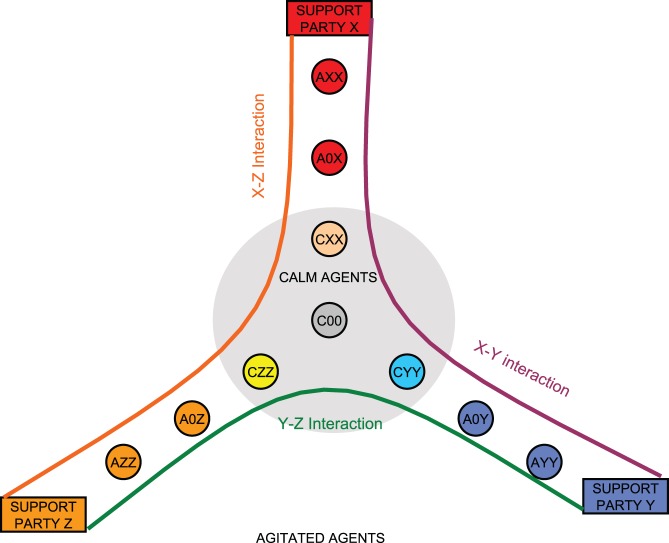
Graphical representation of the three-party model. Any communication between a pair of agents will result in the bipartisan dynamics described in Table 1, and therefore can be described in the seven states of the E/I/O model with the appropriate choice of the two involved parties, for example the case of agent CZZ receiving the message A0Y. The ‘true neutral’ C00 state is common for all interactions. It is therefore possible to derive a multiple-choice dynamics from the binary system introduced in [36].

For the purpose of the current simulations, it turned out that we must distinguish between the ‘true neutrals’ (people with no information nor opinion, C00 state) and left-wing supporters, changing the whole model from binary to three-party simulation. Extending the binary E/I/O approach introduced in [36] to more than two opinions has been facilitated by the fact that the model depends on interactions between pairs of users. It is possible to introduce the third party support, denoted as CZZ, A0Z and AZZ, following the same naming scheme as for the two-party model. As any user-to-user communication involves only two opinions at most, it is possible to use the transition table (Table 1) with appropriate party substitutions. This is graphically presented in Figure 3: each situation may be treated as a pairwise X-Y, X-Z or Y-Z interaction.

Using the three party approach we were able to complement the social network simulations with results covering the agent and message statistics. They are presented in Tables 5 and 6. The results are grouped for the two forum interfaces (OLD and NEW). Each forum was simulated 25 times and the model results are provided with standard deviation, resulting from differences in realization of the network and communication process, as well as from differences in the distribution of initial political sympathies. The ‘observed’ values of political support do not contain the data on the sympathizers of SLD/RP, because such agents were, at the time of the data analysis, which focused on the PO/PiS struggle, grouped together with the true neutrals. One should note that it has been possible to reproduce quite well the intent of the messages (Table 6) and the final average value of the emotions. For the OLD interface, this final calm/agitated ratio is very different from the initial value 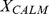 that served as the input parameter: this is the result of interactions between the users. The best fit values of 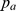 and 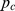 are quite different in the OLD and NEW cases. On the other hand, the best fit probabilities of disagreement and agreement (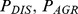) are very close in both cases. That the probability of writing a provocative message by an agitated agent is much higher for the NEW interface is in agreement with expectations.

**Table 5:** Comparison of the observed and simulated agent characteristics for the OLD and NEW fora.

	OLD interface		NEW interface	
Messagecharacteristic	Observed	Simulated	Observed	Simulated
Pro-PO	60%	61.5%±5.1%	71%	46.9%±4.0%
Pro-PiS	25%	19.9%±3.5%	21%	25.6%±2.6%
Pro-SLD/RP		11.0%±1.0%		14.8%±1.6%
Calm	56%	52.4%±5.3%	28%	33.1%±3.1%
Agitated	44%	47.6%±5.3%	72%	66.9%±3.1%

The errors are standard deviation from 25 runs of the simulations for the best fit choice of the model parameters.

**Table 6:** Comparison of the observed and simulated characteristics of the comments posted by the users.

	OLD interface		NEW interface	
Messagetype	Observed	Simulated	Observed	Simulated
Agreement	2348	2608±143	263	423±38
Neutral	4032	4373±337	3817	4580±370
Disagreement	2019	1837±146	663	331±22
Invective	1224	1127±100	508	322±23
Provocation	2561	1979±235	8378	8016±364

The errors are standard deviation from 25 runs of the simulations for the best fit choice of the model parameters.

Generally, the fit of all studied values for the OLD interface, where user quarrels dominate the communication process, is very good. On the other hand, the fit for the new interface is generally worse. We attribute this to the fact that the dominance of single broadcast messages diminishes the communication flow and ‘freezes’ the data on observed participants in their initial status. These are the agents who post one comment and then ‘vanish’ from the forum. There is hardly ground for the simulations of their opinion and emotion changes. Thus, the NEW forum is much more dependent on the input values, for example the calm/agitated average is much closer to the input value of 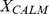. Moreover, the statistics of these ‘one comment participants’ may depend on their motivation to participate, which might be different from the people willing to discuss and quarrel.

## Conclusions

The purpose of the paper was to check the applicability of the E/I/O model into real life environments. The earlier works based on the model used [36], [51] have used simplified connection network topologies (fully connected and short distance square lattice), which could not be compared to the data from observations. In the current paper this has been achieved by using a two-stage agent based modeling. The first stage reproduced the communication network formed by the posting of comments and responses to the comments. The second stage has used such artificial network to study the dynamics of opinions and emotions of the users and the resulting content of the messages.

The model contains a considerable number of parameters, used to fit its results to observations. We recognize this as the major weakness of the approach (as best summarized by the famous quote attributed to John von Neumann “*With four parameters I can fit an elephant, and with five I can make him wiggle his trunk*”). However, our approach was to use parameters that have a clear meaning in psychological/social language, for example the probability of arousal of negative emotions by a contrarian message (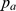), the probability of calming down by a calm message (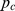), the probability of responding to a contrarian message in a calm or agitated way (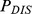) and other parameters used in this work (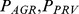). These parameters function within simple, realistic scenarios of agent activities. Eventually, they could be measured in conventional psychological experiments, to provide a test whether the model assumptions are over-simplified or not. Additionally, the separation of the communication and opinion change parts allows a flexible modifications of the model to different social environments.

While the model are not perfect, for example it misses the influence of opinions and emotions on the creation of the communication network, its results are a step in the direction of combining the theoretical, sociophysics approach with the actual social phenomena. Such attempts may eventually build a lasting bridge between the two groups of researchers, over the gap created by the fact of publishing in separate journals, attending separate conferences and using a different language.
